# Effects of sex and retention interval on the retrieval and extinction of auditory fear conditioning

**DOI:** 10.3389/fnbeh.2022.1011955

**Published:** 2022-10-13

**Authors:** Hannah L. Schoenberg, Madeleine Blanchard, Han Yin Cheng, Neil E. Winterbauer, Donna J. Toufexis, Travis P. Todd

**Affiliations:** Department of Psychological Science, University of Vermont, Burlington, VT, United States

**Keywords:** fear conditioning, remote memory, sex differences, freezing, extinction, retrieval

## Abstract

Fear memory retrieval is relevant to psychiatric disorders such as post-traumatic stress disorder (PTSD). One of the hallmark symptoms of PTSD is the repeated retrieval and re-experiencing of the initial fear memory even long after the traumatic event has occurred. Women are nearly twice as likely to develop PTSD following a trauma than men, thus sex differences in the retrieval of fear memories is highly relevant for understanding the development and maintenance of PTSD. In the current study, we aimed to examine sex differences in the retrieval and extinction of either recent or remote fear memories. To do so, we conditioned male and female rats either 1 day (recent) or 28 days (remote) prior to testing retrieval and extinction. While there was no effect of sex or retention interval on initial retrieval, we found that remotely conditioned females exhibited higher rates of freezing than remotely conditioned males in later retrieval/extinction sessions, suggesting a sex difference in the retrieval and/or extinction of remote, but not recent, fear memories. Overall, these results are the first to demonstrate a sex difference in the extinction of remote fear memory, and this may contribute to the differential expression of fear-related disorders like PTSD in men and women.

## Introduction

The ability to form and later retrieve fear memories is highly adaptive. Fear memories promote survival by guiding behavioral responses to avoid potential threats in the future. However, fear memory processes can also contribute to the ontology and maintenance of psychiatric disorders. For example, one of the hallmark diagnostic criteria for post-traumatic stress disorder (PTSD) is chronic re-experiencing of the memory of the traumatic event, even long after the event occurred (National Institute of Mental Health, 2022). The lifetime prevalence of PTSD in the United States is nearly 7% (Kessler et al., 2005); however, women represent a significantly higher proportion of cases, with some reports suggesting that women are two- to three-times more likely to be diagnosed with the disorder than men following a traumatic event (Kessler et al., 2005; Koenen and Widom, 2009). Thus, in order to understand and effectively treat PTSD, it is critical to understand the behavioral and neurobiological differences between males and females with respect to fear memories.

Fear memories are often studied in rodents using contextual fear conditioning. In this procedure, rats or mice are placed in the conditioning apparatus and receive mild foot-shock(s). Re-exposure to the conditioning apparatus, or context, elicits conditioned fear responses (e.g., Fanselow, 1980). Using this procedure, several studies have examined how contextual fear learning and memory may differ between males and females. For example, an early study found that male rats froze more than females when re-exposed to the original conditioning context (Maren et al., 1994). While this finding has been replicated (Pryce et al., 1999; Chang et al., 2009; Poulos et al., 2015; Colon et al., 2018; Russo and Parsons, 2021) there are also contradicting reports in the literature (e.g., Dachtler et al., 2011; Fenton et al., 2014).

In addition to potential differences in the expression of contextual fear, there is some evidence that generalization of contextual fear to a novel context is influenced by sex. For instance, Keiser et al. (2017) found greater degrees of generalization in female mice compared to males, and Asok et al. (2019) reported more generalization in female mice tested 3 weeks after initial conditioning, specifically when testing occurred first in the novel context. Further, using step-through avoidance conditioning, Lynch et al. (2013) demonstrated that males and females have equivalent context discrimination when tested early after conditioning. However, when the retention interval increased to either 5 or 7 days, female rats showed more generalization (greater responding in the second context) than males. Nevertheless, despite the apparent converging evidence of greater generalization in females than males in most studies, there is contradicting evidence that males show stronger generalization of contextual fear to a second context, raising the possibility that some sex differences may be parameter-specific (Colon et al., 2018).

In addition to contextual cues, fear responses can also be elicited by discrete cues that were present in the environment during the aversive event. For example, in Pavlovian fear conditioning, discrete cues (e.g., tones, lights) gain the ability to elicit fear responses through pairings with mild-foot shock. Although there are some exceptions, a general pattern in the literature is that that males and females exhibit relatively similar conditioned fear to discrete cues during retrieval tests [Maren et al., 1994; Voulo and Parsons, 2017; Colon et al., 2018; but see Graham et al. (2009) and Gresack et al. (2009)]. However, the majority of these studies have tested retrieval shortly after conditioning (e.g., within 24 h). Thus, less is known about sex differences in cued fear retrieval when the interval between acquisition and testing is much longer. The use of longer retention intervals may be particularly relevant for studying fear memories in PTSD, as PTSD diagnoses require the presence of memory-related symptoms for at least 1 month (American Psychiatric Association, 2013); additionally, these patients tend to have chronic, recurring symptoms, including persistent, disruptive memories (National Institute of Mental Health, 2022).

The purpose of the present study was to compare male and female rats in the retrieval of cued fear conditioning acquired either recently or remotely. We chose to examine differences between recent and remote memories because it is broadly acknowledged that as memories age, their neurobiological correlates undergo significant reorganization (e.g., systems consolidation; Frankland and Bontempi, 2005). However, whether and/or how these processes differ between females and males, and whether these differences present behaviorally, is yet unknown. One prior study has examined retrieval of cued fear in male and female rats at retention intervals of either 1 or 14 days (Colon et al., 2018), and reported no difference between sexes, although these authors suggested a ceiling effect may have impacted their ability to detect potential effects. The present study therefore extends this prior work in at least two ways. First, to complement the study by Colon et al. (2018), which tested remote memory at a 14-day retention interval, we compared retrieval in groups with retention intervals of either 1- or 28-days. The use of the longer retention interval (28 days) allowed us to assess if sex differences emerge at later time points. Second, all rats received multiple sessions of tone retrieval. The purpose of this was to gradually extinguish fear to the tone, allowing us to assess the impact of sex on fear retrieval/extinction across a broad range of the response scale.

## Materials and methods

### Subjects

The subjects were 63 (31 male, 32 female) experimentally naïve Long-Evans rats (Envigo Laboratories, Indianapolis, IN, USA) 75–90 days old upon arrival. Rats were allowed 1 week to acclimate to the vivarium while housed in pairs. On the first day of behavioral procedures rats were then individually housed with plastic tunnels for enrichment in 12 × 7.5 × 7.5 in plastic caging for the remainder of the experiment. Rats were assigned to one of four groups: Male Remote (*n* = 15), Male Recent (*n* = 16), Female Remote (*n* = 16), and Female Recent (*n* = 16) Food and water were available ad libitum (LabDiet 5P00 Prolab RMH 3000, LabDiet, St. Louis, MO, USA) in a climate-controlled colony room on a 12:12 light-dark cycle. Throughout the experiment, rats were monitored and cared for in compliance with the Association for the Assessment and Accreditation of Laboratory Animal Care guidelines and the University of Vermont Institutional Animal Care and Use Committee.

### Behavioral apparatus

Behavioral procedures occurred in 16 conditioning chambers (Med Associates, Inc., St. Albans, VT, ENV-007; 24 cm W × 30.5 cm L × 29 cm H), which were modified to create 4 sets of distinct “contexts.” All chambers had the following common features. Each chamber was housed in a sound-attenuating cabinet (Med Associates, ENV-017M; 66 cm W × 56 cm L × 56 cm H) outfitted with an exhaust fan to provide airflow and background noise (68 dB). All 16 chambers were outfitted with a food cup, recessed in the center of the front wall, a retracted lever (Med Associates, ENV-112CM), located on the right of the front wall, and an inactive nose-poke aperture (2 cm in diameter) located 3 cm above the food cup. All chambers also had a panel light (Med Associates, ENV-221M) on the right front wall (16 cm above the grid floor), a house light (Med Associates, ENV-215M) centered on the back wall 24 cm above the grid floor, and a speaker (Med Associates, ENV-224AM) located 20 cm above and to the right of the food cup. Only the house light was illuminated throughout the experiment. The speaker was used to deliver a 2000 Hz tone for 10 s (the conditioned stimulus, CS), and the grid floor was used to deliver a 1.0-mA, 1.0-s shock (the unconditioned stimulus, US). Security cameras were mounted to the wall outside each sound-attenuating cabinet, and an 8-cm hole in the chamber wall allowed for video recording from the wall opposite the door.

Sets of four chambers were modified to create four different contexts. For the first distinct context (“Bedding” context), the ceiling and side walls were clear acrylic plastic, the front and back walls were brushed aluminum, and the grid floor was stainless-steel rods (5 mm in diameter) spaced 1.5 cm apart (center-to-center). In addition, approximately 6 oz of woodchip bedding was placed in the tray below the grid floor. For the second set of boxes (“Anise” context), the ceiling and door were covered with laminated black and white checkerboard paper with 3.5 cm black and white squares, and three panels on the back wall were covered in black electrical tape to provide a distinct visual feature. The grid floor was staggered, such that every other bar was on a different plane offset by 0.5 cm, and the tray below the grid floor was painted black. Approximately 5 mL of 10% Anise extract (McCormick, Baltimore, MD, USA) was placed in a plastic dish on the floor directly outside the chamber (inside the cabinet) to the right of the chamber door at the beginning of every session to serve as a distinct olfactory cue.

For the third set of boxes (“Vicks” context), the ceiling and door were covered with wallpaper made from laminated gray construction paper. There was an additional panel light (which remained off) and retracted lever on the left side of the front wall. The floor consisted of alternating stainless-steel rods with different diameters (0.48 and 1.27 cm), spaced 1.6 cm apart from center to center, and the tray beneath the floor was painted gray. Prior to each session approximately 0.5 mL of Vicks VapoRub ointment (Vicks, Cincinnati, OH, USA) was placed in the plastic dish outside the door to chamber. For the fourth set of boxes (“Coconut” context), the ceiling and door were covered with rows of blue dots (3 cm in diameter) that were spaced approximately 1.75 cm apart. There was also an additional panel light (off) and retracted lever on the left side of the front wall. In these chambers the floor consisted of stainless steel rods (5 mm in diameter) arranged such that there was a slight arch in the floor between front and back wall: the highest rod at the center was approximately 1 cm higher than the two rods at either end of the grid floor. A small dish of 10% coconut extract (McCormick, Baltimore, MD, USA) was also placed on the floor to the right of the chamber door.

In the current experiment, each group of rats experienced two contexts, counterbalanced as Context A and B. Half of the rats experienced the Bedding and Anise boxes (counterbalanced as Context A and B) and the other half of the rats experienced the Vicks and Coconut boxes (counterbalanced as Context A and B). Assignment to a particular pair of contexts was counterbalanced across sex and retention interval. Thus, half of the rats in each of the four behavioral conditions (i.e., Male Remote, Male Recent, Female Remote, Female Recent) were trained in the Bedding/Anise pair, and the other half in the Vicks/Coconut pair.

### Behavioral procedures

All behavioral procedures were conducted between 8:00 am and 2:00 pm, and the timing of procedures was kept consistent for each group.

#### Conditioning

**FIGURE 1:**
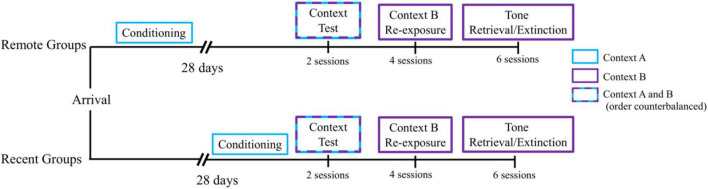
Experimental timeline. All groups received a single session of auditory fear conditioning in Context A, followed by a 28-day retention interval (Remote Groups) or a 1-day retention interval (Recent Groups) before being tested for context fear retrieval in Contexts A and B (order counterbalanced). On the subsequent 2 days, all groups received 2 daily sessions of Context B Re-exposure, for a total of 4 sessions. Next, all groups received 3 daily sessions of tone retrieval/extinction in Context B for 2 days, for a total of 6 sessions.

All rats received a single day of auditory fear conditioning in Context A. Each session consisted of 3 presentations of the CS, a 10-s tone, which terminated with the onset of the US, a 1-mA, 1-s shock. The first trial began 3 min after rats were placed in the chambers. The time between shock and the next CS presentation was 64 s. Subjects remained in the chambers for 90 s after the last trial before being returned to their home-cages. Half of the rats remained in their home cages for a 28-day retention interval. The other half received a 24-h retention interval. As shown in Figure 1, we staggered the start of the experiment (conditioning), so that all rats received the subsequent phases of the experiment on the same day and were therefore the same age at the time of testing.

#### Context tests

Following either a 1- or 28-day retention interval (see Figure 1), all rats were then given a test session in Context A and Context B on the same day, separated by approximately 3 h. During each session, rats were returned to the apparatus for a 4.5-min period in which no tones or shocks were presented and freezing to the context was monitored. The order of testing in A and B was counterbalanced within each group of rats such that half of the rats were tested in Context A first and Context B second, and the other half had the reverse order.

#### Context B re-exposure

Over the course of the next 2 days, rats were exposed to Context B alone for four 20-min sessions (see Figure 1). There were two sessions per day, separated by approximately 3 h. During these sessions, no tones or shocks were presented. The purpose of these sessions was to reduce any generalized fear to the context alone prior to testing tone retrieval.

#### Tone retrieval and extinction

Tone retrieval was tested in Context B. Each session consisted of 30 presentations of the tone with no shocks presented (64 s ITI). The first trial began 3 min after rats were placed in the chamber, and rats were removed from the chamber following the last CS presentation. For two consecutive days (see Figure 1), there were three sessions per day, separated by approximately 1.5 h. Thus, there were a total of six sessions of tone retrieval/extinction.

### Estrous cycle monitoring

In order to monitor the estrous cycle, vaginal smears were collected from all female rats (both Recent and Remote groups) for 4 days prior and 4 days after conditioning for the Remote group, and again starting 4 days prior to conditioning for the Recent group, continuing through the end of the experiment. Smears were collected by inserting a cotton swab dampened with distilled water less than a centimeter into the vaginal canal (to avoid inducing pseudopregnancy) and rolling the tip against the vaginal wall. Samples were then transferred to dry glass slides. All samples were taken each day between 11:45 am and 1:30 pm. Following the end of the experiment, all slides were stained using 0.1% Crystal Violet stain (e.g., McLean et al., 2012), cover-slipped, and evaluated under a light microscope at 10X objective. Number, proportion, and type of cells (nucleated epithelial cells, cornified epithelial cells, leukocytes, and neutrophils) were used to determine if rats fell within one of four stages: proestrus, estrus, metestrus, or diestrus (Cora et al., 2015; Hilz et al., 2019). All samples were evaluated by two trained observers.

### Behavioral observations and data analysis

Freezing was the main dependent measure, defined as total motor immobility except for breathing (Blanchard and Blanchard, 1969; Fanselow, 1980). For the conditioning session, freezing is reported during the 64-s period before the first trial (baseline freezing) and during each of the 10-s tones (CS freezing). In addition, during the conditioning session, we measured activity bursts to the 1-s foot shocks and a control period of 1-s prior to the first CS as a measure of shock reactivity. Shock reactivity was assessed so as to account for any group differences in the experience of shock that could potentially influence differences in learning as measured in later tests (e.g., Wiltgen et al., 2001). Rat position data was collected from every frame of the video data by a trained observer (who clicked on the target point on each randomly presented video frame), smoothed using a three frame rolling average to reduce jitter, and summarized by adding the distances (in normalized pixels) between these points over all pairs of frames during each period. During the context test sessions as well as the re-exposure sessions in Contexts A and B, freezing is reported for the first 4.5 min of each session. During the tone retrieval/extinction sessions, freezing is reported for the 64-s period prior to the first tone (pre-CS period), as well as during tone presentations.

Automated scoring of freezing was conducted using the following method: video streams were acquired in near-infrared (720P resolution, 29.97 frames per second) by Anpviz IPCameras (model IPC-B850W) mounted in each chamber. Streams were delivered over a dedicated ethernet network and captured by a computer running ffmpeg. Recordings were subsequently scored by first computing the absolute difference in pixel intensity at every pixel on each pair of subsequent frames. A per-frame activity measure was produced by averaging this difference over all pixels. Inspection of the distribution of (log10-transformed) activity scores revealed a clear bimodal distribution of activity, with the mode of the lowest scores reflecting video noise and mode of the higher scores reflecting rat movement. These distributions varied almost solely by chamber/camera. Presumptive freezing was therefore defined as occurring, on a per-chamber basis, when the activity score fell below the value visually marking the beginning of the rat-movement related portion of the distribution. Activity scores were then averaged in 1 s bins, and only 1 s bins that fell below the threshold were defined to represent freezing [approximating procedures used by the Fanselow laboratory, e.g., Fanselow et al. (2019)]. Algorithmically scored freezing correlated well with freezing scored by trained human observers, with all *R*-values exceeding 0.80. Tone retrieval/extinction videos were further screened to exclude immobility due to sleeping, boredom, or fatigue from the freezing measure. A trained observer watched video from every CS presentation for each rat, and trials where the animal was clearly sleeping, in a sleep-related posture, or otherwise displaying no fear-related behavior were manually marked as non-freezing. Classification of a small subset of more ambiguous trials was confirmed by a second observer, yielding the final adjusted scores. Freezing data were statistically analyzed using between subjects analysis of variance (ANOVA) and repeated measures ANOVA where appropriate.

In addition to freezing behavior, this is some evidence that female rates engage in other, escape-like responses (e.g., “darting,” flight; Gruene et al., 2015; Greiner et al., 2019) more than males. Because higher performance of these alternate responses can potentially result in the misattribution of lower freezing in females as lower fear (Gruene et al., 2015), we examined the presence of darting during the six tone retrieval/extinction sessions in males and females. The same videos used to score freezing were used to score instances of darting during all of the 10-s tones in all sessions. Darting was defined and scored as “rapid, forward movement across the chamber that resembled an escape-like response” (Gruene et al., 2015).

## Results

### Estrous cycle

While the estrous cycle was monitored throughout the duration of the experiment, adequate analyses based on phase were not possible given that sample sizes for individual phases and/or low vs. high hormone phases were too underpowered (e.g., during tone retrieval/extinction sessions 1–3, only 5 out of the 31 females were in the proestrous phase) to reliably detect a statistical influence of estrous phase.

### Conditioning

#### Freezing

**FIGURE 2:**
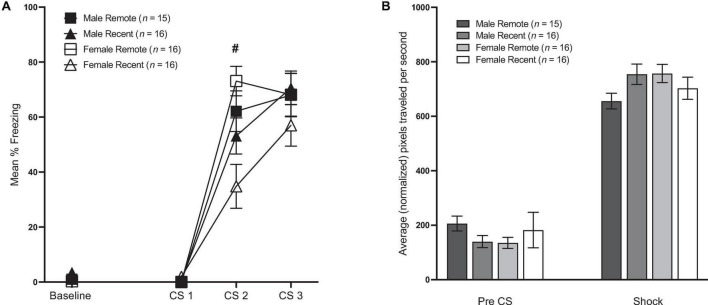
**(A)** Mean percent freezing (±SEM) during conditioning. Baseline represents the 64-s baseline period preceding the first tone. CS 1–3 = the 10-s tones that preceded shock. There was a small but significant difference in freezing during the baseline period, such that Remote groups froze less than Recent groups. During conditioning, Recent groups froze significantly less than Remote groups during CS 2 (# indicates a significant difference between Recent and Remote, *p* < 0.05), but there were no differences in freezing during CS 1 or CS 3 (see Section “Results” for details). **(B)** Shock reactivity during conditioning. Average movement in the 1-s before the first shock [Pre-CS (±SEM)] and average movement across the three 1-s shocks (±SEM).

Mean percent freezing during conditioning is presented in Figure 2A. Freezing during the baseline period was analyzed with a 2 (sex: male vs. female) × 2 (retention interval: 1 vs. 28 days) ANOVA (for means and standard errors, see Table 1). Unexpectedly, there was a significant main effect of retention interval [*F*_(1,59)_ = 7.20, *p* = 0.009, η_*p*_^2^ = 0.109], with remotely conditioned rats exhibiting slightly lower percent freezing (*M* = 0.65, *SD* = 1.25) than recently conditioned rats (*M* = 2.64, *SD* = 3.97) during the 64-s baseline period. Although significant, the difference between means was remarkably small (∼2%). No other significant differences were observed between groups.

**TABLE 1:** Mean percent freezing (±SEM) during baseline periods for each group during conditioning and tone extinction/retrieval sessions.

Conditioning		Tone extinction/retrieval					
Group		1	2	3	4	5	6
Male remote	1.03 (0.39)	3.28 (1.09)	15.23 (3.66)	32.72 (7.27)	44.00 (6.92)	32.92 (9.03)	29.03 (6.38)
Male recent	3.46 (1.26)	4.10 (1.00)	27.87 (8.10)	31.49 (7.22)	57.54 (8.96)	25.54 (6.49)	29.03 (7.07)
Female remote	0.29 (0.21)	10.76 (4.19)	22.91 (5.23)	45.74 (8.36)	32.00 (5.73)	30.67 (6.38)	37.74 (6.87)
Female recent	1.83 (0.56)	8.41 (3.48)	25.05 (5.96)	28.00 (5.28)	33.23 (6.68)	31.08 (5.73)	31.49 (5.09)

Freezing during the CS presentations in the conditioning session was analyzed with a 2 (sex: male vs. female) × 2 (retention interval: 1 vs. 28 days) × 3 (CS presentation) repeated-measures ANOVA. This revealed a significant effect of CS presentation [*F*_(2,118)_ = 185.56, *p* < 0.001, η_*p*_^2^ = 0.759]. Additionally, there was a significant main effect of retention interval [*F*_(1,59)_ = 4.77, *p* = 0.033, η_*p*_^2^ = 0.075] as well as significant CS presentation × retention interval interaction [*F*_(2,118)_ = 6.42, *p* = 0.002, η_*p*_^2^ = 0.098]. Follow-up comparisons to further analyze this interaction revealed no differences between groups during the first CS presentation [*F*_(1,59)_ = 1.55, *p* = 0.218]. While the Recent group froze significantly less than the Remote group during the second CS presentation [*F*_(1,59)_ = 11.77, *p* = 0.001, η_*p*_^2^ = 0.166], the difference was fleeting, as levels of freezing were equivalent by the third and final CS presentation [*F*_(1,59)_ = 0.33, *p* = 0.566]. There was no main effect of sex (*p* = 0.426) nor sex × retention interval interaction (*p* = 0.092). Neither the CS × sex (*p* = 0.59), nor the CS × sex × retention interval interaction were significant (*p* = 0.116).

#### Shock activity

Burst activity during the pre-CS period and during the shocks can be seen in Figure 2B. Total movement during the pre-CS period was analyzed with a 2 (sex) × 2 (retention interval) ANOVA. As expected, movement during the pre-CS period did not differ by sex or by retention interval (*p*’s > 0.05). Average movement during the shocks was analyzed in a 2 (sex) × 2 (retention interval) × 3 (shock periods) ANOVA. While neither the main effect of sex nor the effect of retention interval was significant [*F*_(1,59)_ = 0.49, *p* = 0.486; *F*_(1,59)_ = 0.39, *p* = 0.534, respectively], there was a significant sex × retention interval interaction [*F*_(1,59)_ = 4.64, *p* = 0.035, η_*p*_^2^ = 0.073]; however, pairwise comparisons revealed that group differences were only marginal {Male Remote < Female Remote [*F*_(1,59)_ = 4.01, *p* = 0.050]; Male Remote < Male Recent group [*F*_(1,59)_ = 3.80, *p* = 0.056]}.

### Context tests

#### Freezing

**FIGURE 3:**
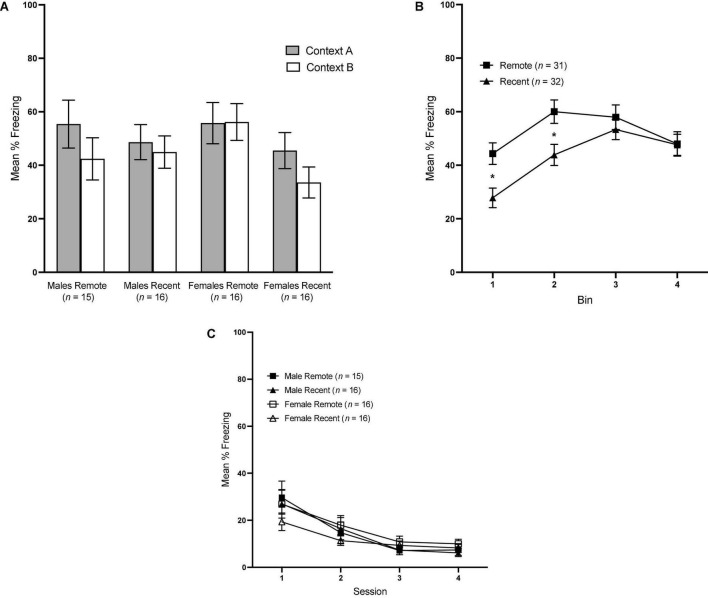
**(A)** Mean percent freezing (±SEM) in Context A vs. Context B for all groups during the context retrieval test. **(B)** Mean percent freezing (±SEM) during the context tests across 4 1-min bins, collapsed across context and sex. **(C)** Mean percent freezing (±SEM) during sessions 1–4 of Context B Re-exposure. *Indicates significant with *p* < 0.05.

Overall mean freezing in Contexts A and B during the context tests is presented in Figure 3A. Surprisingly, freezing was not significantly higher in context A than B, regardless of experimental group: a repeated-measures ANOVA revealed a lack of significant effects of context [*F*_(1,59)_ = 3.33, *p* = 0.073], retention interval [*F*_(1,59)_ = 2.43, *p* = 0.124] or sex [*F*_(1,59)_ = <0.001, *p* = 0.987].

To further probe contextual fear retrieval, we also examined freezing across 4, 1-min bins during the context tests with a 4 (bin: 1–4) × 2 (context: A vs. B) × sex × retention interval repeated-measures ANOVA. There was a significant main effect of bin [*F*_(3,177)_ = 20.82, *p* < 0.001 η_*p*_^2^ = 0.261] and bin × retention interval interaction [*F*_(3,177)_ = 5.03, *p* = 0.002, η_*p*_^2^ = 0.079]. No other main effects or interactions reached significance. Freezing in the Recent and Remote groups across bins is presented in Figure 3B, collapsed across sex and context, since neither were significant. Pairwise comparisons revealed a significant difference between Recent and Remote groups at bin 1 [*F*_(1,59)_ = 7.062, *p* = 0.010, η_*p*_^2^ = 0.107] and bin 2 [*F*_(1,59)_ = 5.44, *p* = 0.023, η_*p*_^2^ = 0.084]. In both instances, Remote groups froze significantly more than Recent groups, indicating that, in the first half of the context tests, Remote groups demonstrated higher levels of freezing than Recent groups.

### Context B re-exposure

Mean percent freezing across the four sessions of Context B re-exposure is shown in Figure 3C. A 4 (session) × 2 (sex) × 2 (retention interval) repeated-measures ANOVA revealed a significant effect of session, *F*_(3,177)_ = 31.0, *p* < 0.001, η_*p*_^2^ = 0.344, and no other significant factors, indicating that extinction of any generalized fear to Context B proceeded equivalently in all four groups.

### Tone retrieval/extinction

**FIGURE 4:**
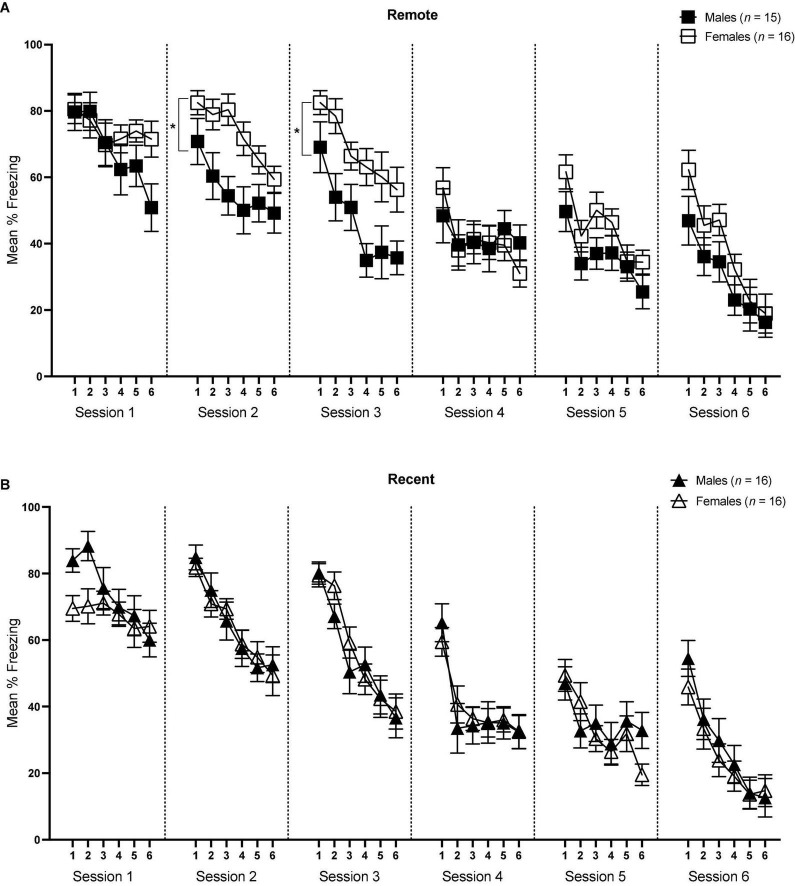
Mean percent freezing (± SEM) during the tone retrieval/extinction tests in the Remote [upper panel **(A)**] and Recent [lower panel **(B)**] groups across blocks of 5 trials during each session. *Indicates significant with *p* < 0.05.

Mean percent freezing across sessions of tone retrieval/extinction can be seen in Figure 4A (Remote Groups) and Figure 4B (Recent Groups). Baseline levels of freezing were compared for each session in a 2 (sex) × 2 (retention interval) ANOVA. Freezing during the tone within each session was analyzed in separate 6 (5-trial blocks) × 2 (sex) × 2 (retention interval) repeated-measures ANOVAS.

For session 1, ANOVA revealed a significant effect of block [*F*_(5,295)_ = 14.46, *p* < 0.001, η_*p*_^2^ = 0.197] and significant block × sex interaction [*F*_(5,292)_ = 5.30, *p* < 0.001, η_*p*_^2^ = 0.082]. Importantly, there was no significant difference in the first 5-trial block between males and females [*F*_(1,59)_ = 2.45, *p* = 0.124], suggesting that initial tone-shock retrieval did not differ between the sexes. However, sexes did vary slightly at other points in the session—males appeared to freeze more in block 2, although this was marginally significant (*p* = 0.050), and females froze more in block 6 (*p* = 0.032). Of particular interest was the change in freezing rates across the session in both sexes. To address this, an ANOVA assessing freezing rates over blocks was conducted separately for males and females. These analyses indicated that while males showed a significant decline in freezing across the session [*F*_(5,145)_ = 22.01, *p* < 0.001, η_*p*_^2^ = 0.432], there was no significant change in levels of freezing in females [*F*_(5,150)_ = 1.11, *p* = 0.358]. Furthermore, there were no main effects of sex [*F*_(1,59)_ < 0.001, *p* = 0.985], retention interval [*F*_(1,59)_ < 0.001, *p* = 0.996], or sex × retention interval interaction [*F*_(1,59)_ = 2.28, *p* = 0.136]. Finally, freezing during the baseline period did not significantly differ between males and females or Recent and Remote groups (*p*’s > 0.05). Overall, results from session 1 indicate that initial tone retrieval was not affected by sex or retention interval, but that as the session progressed, males began to exhibit initial extinction (reduction in freezing) of auditory fear, while females maintained continued high levels of freezing to the tone.

For session 2, the effects of block and sex were significant [*F*_(5,295)_ = 34.09, *p* < 0.001, η_*p*_^2^ = 0.366; *F*_(1,59)_ = 4.47, *p* = 0.039, η_*p*_^2^ = 0.07, respectively]. While the main effect of retention interval was not significant [*F*_(1,59)_ = 0.003, *p* = 0.957], there was a significant sex × retention interval interaction [*F*_(1,59)_ = 4.88, *p* = 0.031, η_*p*_^2^ = 0.076]. Follow-up pairwise comparisons revealed that females froze significantly more than males within the Remote condition [*F*_(1,59)_ = 9.20, *p* = 0.004, η_*p*_^2^ = 0.135], whereas there was no difference in degree of freezing between males and females within the Recent condition (*p* = 0.946). There were no significant differences in baseline freezing levels (*p’s* > 0.05). Thus, while freezing significantly declined across blocks in all groups, freezing was comparable between males and females in the Recent condition, but differed significantly between sexes in the remote condition, with females maintaining a higher level of freezing than males across the session.

The sex difference in the Remote condition that was observed in session 2 continued in session 3. There was a significant main effect of block [*F*_(5,295)_ = 41.99, *p* < 0.001, η_*p*_^2^ = 0.416] as well as sex [*F*_(1,59)_ = 8.48, *p* = 0.005, η_*p*_^2^ = 0.126]. The main effect of retention interval was not significant [*F*_(1,59)_ = 0.09, *p* = 0.766], though there was a significant sex × retention interval interaction [*F*_(1,59)_ = 5.44, *p* = 0.023, η_*p*_^2^ = 0.084]. As in session 2, in session 3 the Female Remote group froze significantly more than the Male Remote group [*F*_(1,59)_ = 13.53, *p* = 0.001, η_*p*_^2^ = 0.186], and again, this effect was not seen in the recently conditioned groups [*F*_(1,59)_ = 0.17, *p* = 0.681]. There were no significant differences in baseline freezing levels (*p’s* > 0.05).

In session 4, there was a significant main effect of block [*F*_(5,295)_ = 15.79, *p* < 0.001, η_*p*_^2^ = 0.211], suggesting that freezing declined across the session, though a lack of any other significant effects indicated that all groups reduced freezing comparably. Baseline responding was significantly higher in males than females [*F*_(1,59)_ = 6.02, *p* = 0.017, η_*p*_^2^ = 0.093], though this difference did not appear in freezing during the tones.

Similarly, no significant differences were observed between groups in either session 5 or session 6. The only significant effect in each session was that of block [Session 5: *F*_(5,295)_ = 16.48, *p* < 0.001, η_*p*_^2^ = 0.218; Session 6: *F*_(5,295)_ = 30.08, *p* < 0.001, η_*p*_^2^ = 0.392]. There were no significant differences in baseline freezing in either session between groups (*p*’s > 0.05). Together, these results suggest that freezing reliably declined across later sessions of extinction, though after session 3 the effects of sex and retention interval were no longer present.

**FIGURE 5:**
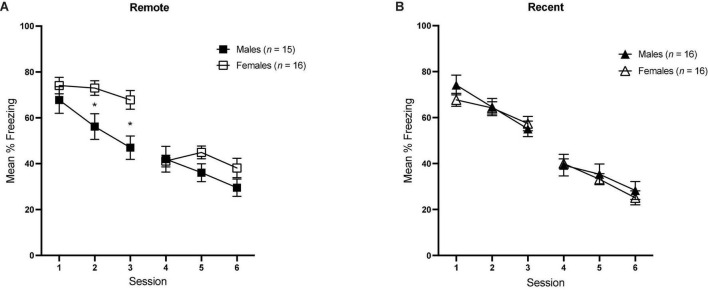
Overall mean percent freezing (±SEM) during sessions of tone retrieval/extinction in the Remote [**(A)** left] and Recent [**(B)** right] groups. *Indicates significant with *p* < 0.05.

In order to examine the progression of extinction across sessions between groups, we also examined mean percent freezing during tone presentations for each session, averaged over all 30 CS presentations [see Figure 5A (Remote groups) and Figure 5B (Recent groups)]. Average freezing during each session was analyzed with a 6 (Session) × 2 (sex) × 2 (retention interval) repeated-measures ANOVA.

There was a significant main effect of session [*F*_(5,295)_ = 178.77, *p* < 0.001, η_*p*_^2^ = 0.059] as well as a significant sex × session interaction [*F*_(5,395)_ = 3.67, *p* = 0.003], Furthermore, the sex × retention interval × session interaction approached significance [*F*_(5,295)_ = 2.12, *p* = 0.063], which is consistent with the individual within-session analyses conducted previously: in session 2, the sex × retention interval interaction was significant [*F*_(1,59)_ = 4.88, *p* = 0.031, η_*p*_^2^ = 0.076], and the same was true in session 3 [*F*_(1,59)_ = 5.44, *p* = 0.023, η_*p*_^2^ = 0.084]. As previously described, the higher overall freezing observed in females is driven by the sex difference in the Remote condition, specifically.

There is some evidence that, in addition to freezing, rats exhibit a darting response to conditioned fear cues, and that female rats do so at higher rates than males. In order to assess darting, we manually scored the presence of rapid, escape-like movements across the conditioning chamber during the 10 s tone for all rats across all tone presentations during extinction/retrieval sessions. We observed a single instance of darting, exhibited by one Female Recent rat during session 1; no other instances were observed. Thus, there was no discernable difference between males and females in this measure in the current study.

## General discussion

Understanding sex differences in fear conditioning is critically important for appropriate treatment of many psychopathologies that involve dysregulated fear learning, such as PTSD. The purpose of this study was to compare retrieval of auditory fear conditioning between male and female rats that were tested at either a recent (1 day) or remote (28 days) time-point. Our results are relevant to both initial tone retrieval and subsequent extinction. We observed no group differences in retrieval of auditory fear during the early portion of session 1 [see also Colon et al. (2018)]. In contrast, during sessions 2 and 3, we observed a marked sex difference in freezing during the tone, with females freezing more than males—but only in the Remote groups; no sex difference was observed in the Recent Groups.

During initial conditioning we observed group differences that were not anticipated: there was a small but significant difference in baseline freezing between Recent and Remote groups (Remote groups showed lower freezing at baseline), while Recent groups exhibited significantly lower freezing during the second tone than Remote groups. It should be noted that, while all testing for context and tone retrieval/extinction occurred when rats were the same age, conditioning occurred when Recent groups were a few weeks older than Remote groups. The effects of age on fear conditioning are most frequently attributed to conditioning that occurs in adolescence vs. adulthood (e.g., Toledo-Rodriguez and Sandi, 2007), and both the Recent and Remote groups were fully adult when conditioning took place, therefore the potential influence of age is not clear. In addition, conditioning occurred on different days for these groups, and there may have been environmental factors (e.g., noise or activity in the vivarium on the days around conditioning) that contributed to differences in freezing. We note that the difference observed between the Recent and Remote groups during the conditioning session was only present on trial 2, and that by trial 3 there were no differences in overall freezing levels. As noted, during sessions 2 and 3 of tone retrieval/extinction, we observed a sex difference in the Remote condition, and not the Recent condition. Because there were no sex differences observed during initial conditioning, it seems unlikely that the recent vs. remote difference observed during conditioning fully explains the results observed during later tone testing.

Our analysis of the first session of tone retrieval/extinction showed that initial retrieval did not differ based on sex or retention interval (see Figures 4, 5). A study by Colon et al. (2018) tested tone retrieval in males vs. females either 1 or 14 days after auditory conditioning and found similar results: retrieval did not differ between sexes at either retention interval. Our findings are complementary and extend this research to suggest that sex differences do not emerge even at later time-points after conditioning (here, 28 days). Colon et al. (2018) acknowledged that freezing may have been at a ceiling in their study, making sex differences difficult to detect. This was not the case in the present study, as both sexes were at approximately 75% mean freezing at the onset of testing.

The primary finding from our study was that, despite initial retrieval being relatively equivalent between sexes, a dissociation emerged between recent and remote memories that was sensitive to sex: remotely conditioned females showed higher levels of freezing in sessions 2 and 3 relative to their male counterparts, while there was no difference between recently conditioned males and females throughout the entirety of tone extinction/retrieval. One possibility is that the excitatory tone-shock association was more strongly encoded/consolidated in females in the Remote group, resulting in stronger resistance to extinction in those rats. Indeed, there are several lines of research indicating sex differences in the neurobiological processes involved in the consolidation of fear memory (e.g., Devulapalli et al., 2021; Farrell et al., 2021; Florido et al., 2021; Crestani et al., 2022), largely focusing on protein signaling within the amygdala. Alternatively, it may be that the original fear memory was encoded similarly, but extinction proceeded differentially between the sexes. The results in the Female Remote group are consistent with prior studies that have shown that females exhibit slower extinction following cued fear conditioning compared to males [e.g., Baran et al., 2009; Fenton et al., 2016; Greiner et al., 2019; but see Voulo and Parsons (2017)]. However, these studies used relatively short retention intervals (e.g., 1 day) and still observed this sex difference, whereas we did not see any differences in extinction between recently conditioned male and female groups [see also Gruene et al. (2015), Voulo and Parsons (2017), and Binette et al. (2022)]. Overall, our results are the first to a show sex-specific effect in extinction of remote fear memory.

One of the limitations of our study was that we did not see differential retrieval in the fear-conditioning context (A) vs. the neutral context (B). This was an unexpected result of this study: our laboratory has previously observed differential responding across contexts with other behavioral paradigms using very similar arrangements of contextual cues (e.g., Tavakkoli et al., 2020). Nevertheless, it is interesting to note two factors of the context test that we did observe. Firstly, we found that Remote groups showed higher freezing in the first half of the test than the Recent Groups, which is consistent with previous reports of stronger fear following longer retention intervals (e.g., Poulos et al., 2016). Greater freezing for the Remote groups could reflect incubation of fear, or it could reflect weaker conditioning in the Recent groups. Secondly, we did not detect any sex differences in context retrieval, which is consistent with some of the literature regarding context fear [but see, e.g., Maren et al. (1994), Poulos et al. (2015), and Russo and Parsons (2021)]. For example, Dachtler et al. (2011) observed similar freezing between male and female wild-type mice when they returned to the conditioning context after a 24-h retention period. Additionally, Kosten et al. (2006) observed equivalent freezing between male and female rats during a context test that occurred 24-h after tone-fear conditioning. Thus, it is not unprecedented that male and females show equal contextual fear.

Previous studies have indicated that female rats have a higher propensity than males to exhibit darting, an escape-like response to fear cues (Gruene et al., 2015; Colom-Lapetina et al., 2019; Mitchell et al., 2022). However, in the current study, this behavior was not present; only one instance of darting was observed throughout tone retrieval/extinction [see also Colon et al. (2018)]. It is possible that specific conditioning parameters are necessary to observe this behavior: darting behavior increases with more CS-US pairings during conditioning, initially emerging between 5 and 7 CS-US pairs (Gruene et al., 2015; Mitchell et al., 2022) and commonly when milder foot-shock US’s are used (less than 1 mA; Mitchell et al., 2022). These differ from the parameters used in this study (3 CS-US pairings, 1 mA shock). Alternatively, unlike freezing, darting is not a conditioned behavior but rather occurs as a result of non-associative processes [see Trott et al. (2022)].

While our study was not adequately powered to investigate the role of gonadal hormones in the sex difference we observed [see also Voulo and Parsons (2017)], previous work has indicated that gonadal hormone state, such as phase of estrous or menstrual cycle, can influence multiple aspects of fear learning and memory [for reviews, see Dalla and Shors (2009), Maeng and Milad (2015), Ramikie and Ressler (2018), and Velasco et al. (2019)]. While low vs. high hormone state has previously been shown to have no effect on the acquisition of freezing to discrete cues (Milad et al., 2009; Carvalho et al., 2021), the literature is mixed as to how hormone state interacts with contextual fear learning; some reports indicate facilitated freezing to the context when estrogen and progesterone are high (e.g., Jasnow et al., 2006) where others show the opposite effect (e.g., Gupta et al., 2001) and still others show a lack of any effects of hormone state (e.g., Chang et al., 2009).

Relevant to our study were findings from a study by Milad et al. (2009), in which authors analyzed sex differences and the effect of relative hormone state (low vs. high) during extinction learning and extinction retention (performance in an extinction test 24 h after the initial test). These authors reported that sex differences only emerged when hormone state was taken into consideration. Importantly, they found that hormone state primarily influenced extinction recall rather than initial extinction learning: females in a high hormone state during the initial extinction learning showed lower freezing the following day (thus, better extinction recall) than those in the low hormone state, and hormone state on the extinction recall day did not appear to matter. Given these findings, it is important to note that hormone state may play a role in the sex difference in the extinction of remote fear observed in the current study, but due to the uneven distribution of females in different phases throughout the experiment, we were unable to detect any effect statistically.

In summary, the present experiment extends prior work investigating sex differences in recently vs. remotely acquired memory (Colon et al., 2018). Our results regarding initial retrieval were complementary to those previously reported: we saw no meaningful differences between males and females regardless of the age of the fear memory during the initial trials of tone retrieval. However, we did observe a sex difference following with the longer retention interval during extinction, with the Female Remote group retaining higher levels of freezing (and thus showing greater resistance to extinction) than the Male Remote group. There are a number of possible mechanisms for this difference that have yet to be empirically examined, including sex differences in fear memory consolidation as well as the role of hormone state. Overall, the present study suggests a dissociation between recent and remote memories which is at least in part sensitive to sex, and this may provide a basis for further research into sex-specific mechanisms of fear-related disorders.

## Data availability statement

The raw data supporting the conclusions of this article will be made available by the authors, without undue reservation.

## Ethics statement

This animal study was reviewed and approved by University of Vermont Institutional Animal Care and Use Committee.

## Author contributions

All authors listed have made a substantial, direct, and intellectual contribution to the work, and approved it for publication.
